# Editorial: Brain health across the lifespan: nutritional interventions and physical exercise for healthy aging

**DOI:** 10.3389/fnut.2026.1913073

**Published:** 2026-07-22

**Authors:** Bruce A. Watkins, Andrew C. Shin, Chwan-Li Shen

**Affiliations:** 1 Department of Nutrition, University of California, Davis, Davis, CA, United States; 2 Neurobiology of Nutrition Laboratory, Department of Nutritional Sciences, College of Health and Human Sciences, Texas Tech University, Lubbock, TX, United States; 3 Human Molecular Aging Center, Texas Tech University, Lubbock, TX, United States; 4 Center for Translational Neuroscience and Therapeutics, Lubbock, TX, United States; 5 Center for Excellence in Integrated Health, Texas Tech University Health Sciences Center, Lubbock, TX, United States

**Keywords:** brain plasticity, cognitive aging, dietary intervention, flavonoid, neurodegeneration, nutritional neuroscience, physical exercise

This Research Topic of reviews and original research articles focus on the brain, nutrition, and exercise for healthy aging. Several nutrients and physical exercise appear to help prevent/mitigate aging diseases of the brain. As the brain ages, it undergoes changes in metabolism, plasticity, and cognitive function, making it vulnerable to age-related decline. Growing evidence suggests that diet, including nutrients and plant-derived bioactives, play a critical role in supporting brain health and mitigating these changes. Nutritional components such as omega-3 polyunsaturated fatty acids (PUFA), antioxidants, and polyphenols have shown potential in promoting neuroplasticity and delaying cognitive decline (1). This Research Topic aims to explore the influence of diet and exercise on brain aging, offering insights into dietary strategies that support long-term cognitive health and resilience against neurodegenerative diseases. The focus of this Research Topic was to highlight the following subthemes. 1. The physiological mechanisms linking nutrition to brain aging and cognitive decline. 2. The action of plant-derived bioactive compounds and their effects on neuroplasticity and brain metabolism. 3. Dietary interventions in promoting cognitive resilience against age-related diseases. 4. The synergistic effects of exercise and nutrition on brain health during aging. 5. The role of specific nutrients, such as antioxidants, omega-3 fatty acids, and polyphenols, mitigating brain senescence. 6. The relationship between diet and gut-brain connections. 7. The potential of personalized nutrition to optimize brain function and delay cognitive aging.

By advancing our understanding of how diet, nutrients, and bioactives influence brain aging and cognitive function (2), this Research Topic aims to inform innovative dietary strategies that promote lifelong brain health and resilience against age-related neurodegenerative conditions. With respect to omega-3 PUFA, the literature is replete with fundamental roles in structure function actions of the long-chain n-3 PUFA in brain (3, 4). Thus, this Research Topic of reviews and original research articles focus on the brain, nutrition, and exercise for healthy aging. The brain undergoes changes in nutrient metabolism, neuroplasticity, and cognitive function as we age (1). Several nutrients and physical exercise help prevent and mitigate aging diseases of the brain (2).

According to Chen et al., the neuroprotective synergy of vitamin D and exercise is emerging evidence suggesting complementary benefits on aging-related neuroplasticity and cognitive health. Opportunities exist with at-risk populations, standardized intervention models, lifestyle factors, and mechanistic approaches with vitamers of D on reducing age-related neuropathology and cognitive decline. It is essential in future studies to integrate biological markers (such as BDNF, inflammatory cytokines, oxylipins, and endocannabinoids) (5), and exercise with neuroimaging measures (e.g., functional connectivity and hippocampal volume) for brain health (6, 7).

Tocotrienols likely support cognition-enhancing benefits in healthy adults. In the clinical trial performed by Lopresti et al., adults administered a placebo or 100 mg of tocotrienols daily were monitored for changes in blood biomarkers to access inflammation, oxidative stress, and neurotropic activity. Blood measurements showed statistically significant between-group differences in levels of TNF-α (*p* = 0.043) and hs-CRP (*p* = 0.039). The group supplemented with tocotrienols demonstrated a greater improvement in overall memory (TOMAL-2 CMI) compared to the placebo group. This study provides evidence for the beneficial effect of tocotrienol supplementation for 12 weeks on non-verbal memory in adults with subjective memory complaints.

In another clinical trial run by Kim et al., after a 12-week intervention, subjects that consumed a nutritional supplement silkworm pill (rich in vitamin A and choline) showed significant improvement in delayed memory compared with the placebo group given a pill with the equivalent protein level. No differences were observed in other cognitive domains or secondary outcomes, such as nutrient intake, blood tests, nutrition quotient, or MIND score adherence. The findings are preliminary, however, the supplement showed benefits in mild cognitive impairment (MCI), such as improvement in delayed memory index. Clinical trials are a likely extension of the research in patients to ascertain efficacy of the supplement for MCI.

A new framework is proposed by Xun and Zeng to study carbohydrates and ketogenic metabolites for neuroprotective strategies in diminishing cognitive dysfunction during diabetes. Carbohydrate restriction at or below 50g/d such as low-carbohydrate ketogenic diets (LCKDs), can improve glycemic index and energy balance. Although LCKDs are reported to protect against cognitive dysfunction via synergism of improved insulin sensitivity and lower neuroinflammation in animal studies, significant findings are lacking in clinical studies. Thus, randomized studies in diabetic populations with cognitive endpoints and mechanistic approaches will improve understanding of ketogenic therapies beyond pre-clinical evidence.

A novel review on gut microbiota and brain aging in African populations suggest diet-related factors that influence gut microbiota composition and risk of dementia. The review was designed by Ortutu et al. to explore the composition of traditional African diets vs. those consumed by Western populations to identify factors in African diets that confer neuroprotection. The future goals are to identify region-specific diets and populations to validate causal links to neurodegenerative diseases. One finding of interest was that the microflora such as *Prevotella* species is associated with African diets of high fibers and fermented food that may likely improve gut and brain health.

Polyphenols and exercise-induced organ crosstalk support brain function with potential health improvements and lifespan extension (1). New evidence suggests the dynamic synergism of polyphenols and exercise in altering cellular events on brain physiology, healthy aging, and senescence (1). As an extension of the current polyphenol research on oxidative stress, inflammation, apoptosis, autophagy, metabolic regulation, and neuroplasticity Mao and Yang discussed as implicating factors contributing to head and neck cancer development, neurodegeneration, and lifespan.

The benefits of probiotics for supporting maternal health during pregnancy are limited (Martín-Peláez et al.). For example, the efficacy of probiotics for preventing maternal mental health disorders during pregnancy and postpartum implications remain unclear. Few studies report symptom reductions, and the heterogeneity in bacterial strains, doses, intervention duration, and measurement tools restrict proper interpretations. What is needed, according to Mart'ın-Peláez et al., are fully characterized and adequately powered RCTs for maternal depression and anxiety issues and that use strain-specific bacterial cultures, suitable doses and time intervals, with proper assessment tools. Such studies should examine prebiotics and synbiotics. Improving our understanding of strain-specific microorganisms would help the research and best impact wellness.

A 24-week community-based, multi-component exercise program designed by Xu et al. safely increased skeletal muscle mass and reduced fat mass in older adults but did not produce measurable improvements for screening-level cognitive measures. Exercise programs improved muscle mass and lowered fat mass and some aspects of cognition, but cognitive function was not evaluated in older adults.

Ergothioneine is a bioactive antioxidant found in mushrooms that appears to have neuroprotective actions in animal models. However, the effects and benefits in humans are not well-known, thus a protocol was established by Shan et al. to ascertain the efficacy of mushrooms in preventing cognitive decline in patients at risk of dementia. The potential is great to apply study findings on mushrooms to clinical and public health practices that reduce dementia and improve cognitive health.

This Research Topic is a step forward to building interest and aligning research priorities for future investigations into brain health, nutrition, and cognition in adults. By advancing our understanding of how diet, nutrients, and bioactives influence brain aging and cognitive function, this Research Topic aims to inform innovative dietary strategies that promote lifelong brain health and resilience against age-related neurodegenerative conditions.
